# *Helicobacter pylori* shows tropism to gastric differentiated pit cells dependent on urea chemotaxis

**DOI:** 10.1038/s41467-022-33165-4

**Published:** 2022-10-05

**Authors:** Carmen Aguilar, Mindaugas Pauzuolis, Malvika Pompaiah, Ehsan Vafadarnejad, Panagiota Arampatzi, Mara Fischer, Dominik Narres, Mastura Neyazi, Özge Kayisoglu, Thomas Sell, Nils Blüthgen, Markus Morkel, Armin Wiegering, Christoph-Thomas Germer, Stefan Kircher, Andreas Rosenwald, Antoine-Emmanuel Saliba, Sina Bartfeld

**Affiliations:** 1 Research Centre for Infectious Diseases, Institute for Molecular Infection Biology, Julius Maximilians University of Würzburg, Würzburg, Germany; 2 Helmholtz Institute for RNA-based Infection Research (HIRI), Helmholtz-Centre for Infection Research (HZI), Würzburg, Germany; 3 Core Unit Systems Medicine, University of Würzburg, Würzburg, Germany; 4 Institute of Pathology, Charité Universitätsmedizin Berlin, Berlin, Germany; 5 Department of General, Visceral, Vascular and Paediatric Surgery, University Hospital of Würzburg, Würzburg, Germany; 6 Institute of Pathology, Julius Maximilian University of Würzburg and Comprehensive Cancer Center Mainfranken, Würzburg, Germany; 7 Si-M/‘Der Simulierte Mensch’, Technische Universität Berlin and Charité–Universitätsmedizin Berlin, Berlin, Germany; 8 Institute of Biotechnology, Technische Universität Berlin, Berlin, Germany

**Keywords:** Pathogens, Bacterial pathogenesis, Cellular microbiology, Stomach diseases

## Abstract

The human gastric epithelium forms highly organized gland structures with different subtypes of cells. The carcinogenic bacterium *Helicobacter pylori* can attach to gastric cells and subsequently translocate its virulence factor CagA, but the possible host cell tropism of *H. pylori* is currently unknown. Here, we report that *H. pylori* preferentially attaches to differentiated cells in the pit region of gastric units. Single-cell RNA-seq shows that organoid-derived monolayers recapitulate the pit region, while organoids capture the gland region of the gastric units. Using these models, we show that *H. pylori* preferentially attaches to highly differentiated pit cells, marked by high levels of GKN1, GKN2 and PSCA. Directed differentiation of host cells enable enrichment of the target cell population and confirm *H. pylori* preferential attachment and CagA translocation into these cells. Attachment is independent of MUC5AC or PSCA expression, and instead relies on bacterial TlpB-dependent chemotaxis towards host cell-released urea, which scales with host cell size.

## Abstract (web summary)

The carcinogenic bacterium *Helicobacter pylori* infects gastric cells. Here, the authors show that *H. pylori* preferentially infects differentiated cells in the pit region of gastric units, and this relies on bacterial chemotaxis towards host cell-released urea, which scales with host cell size.

## Introduction

In terms of prevalence, *Helicobacter pylori* (*H. pylori*) is a very successful human pathogen that colonizes the stomach of ~50% of the global population. Although most infected individuals remain asymptomatic, the persistence of the pathogen and the resulting inflammation are associated with an increased risk of developing gastric disease including peptic ulcer, chronic active gastritis, and gastric adenocarcinoma^1,2^.

*H. pylori* evades the luminal gastric acid by replicating in the mucus layer close to the epithelial cells^3^ or by colonizing the glands^4^. Bacteria can attach to epithelial cells by binding to Lewis antigens, frequently presented by host glycoproteins including mucin 5AC (MUC5AC)^5,6^, CEA Cell Adhesion Molecule 5 (CEACAMs)^7,8^ and β1-integrin^9^. Binding to integrins promotes the expression of a bacterial type 4 secretion system (T4SS), which then translocates a bacterial protein, the cytotoxicity-associated gene A (CagA), into the host cell, where it is phosphorylated by host proteins^10^. CagA translocation can lead to loss of cell polarity, which can allow *H. pylori* to obtain nutrients^11,12^. The bacterial T4SS and its effector CagA are thus considered the key driver of pathogenicity and a major risk factor for the development of gastric cancer^13,14^. Bacterial peptidoglycan and ADP-heptose induce an NF-κB inflammatory response^15,16^, which is thought to further drive tissue destruction and subsequent compensatory proliferation^17^.

The gastric epithelium is organized into flask-like invaginations, the gastric units, which are further organized into the regions pit, isthmus and gland. The pit harbours MUC5AC-expressing pit cells. The isthmus is a region of high proliferation. The gland harbours mucin 6 (MUC6)-positive neck cells, pepsinogen C (PGC)-positive chief cells, H^+^, K^+^-ATPase (ATP4A)-positive parietal cells, and chromogranin A (CHGA)-positive enteroendocrine cells. Cells of the gland can have a life span of several months^18^ while pit cells die within 3–5 days^19,20^. In vivo, it is technically not possible, to monitor adherence and CagA-translocation into the different cell types.

In vitro studies in adult stem cell-derived organoids and organoid-derived monolayers have demonstrated that gland cells mount a strong inflammatory response while the pit cells are relatively refractory to infection^21,22^. However, it remains unclear, whether *H. pylori* has a preference to attach to and translocate CagA into a particular cell type.

The recent advances in single-cell RNA sequencing (scRNA-seq) technologies allow to resolve cellular composition of tissues and organoids^23^. It also allows to decompose host–pathogen interaction at an unprecedented resolution, providing a powerful tool to decipher both cellular identities and their function in the complex and heterogeneous outcome of the infection^24,25^.

Here, we combine the power of human adult stem cell-derived gastric organoids and scRNA-seq to identify the *H. pylori* preferential target cell. ScRNA-seq of 3D gastric organoids and 2D monolayers derived from organoids demonstrate that the former mostly contains cells from the gland region, while the latter contains pit cells and especially a population of highly differentiated pit cells. This population is absent from 3D organoids under standard growth conditions but since adult stem cells have full differentiation capacity, 3D organoids can also be directed to generate the cell type, which is a particularly large and granular cell expressing PSCA, GKN1 and GKN2. *H. pylori* preferentially binds to these highly differentiated pit cells. This preference depends on the chemotaxis of the bacteria towards host urea. As a byproduct of protein metabolism, urea secretion scales with host cell size and so does the binding preference of *H. pylori*. Together, the data shows that *H. pylori* urea chemotaxis leads to preferential binding to particularly large, highly differentiated pit cells.

## Results

### Gastric organoids and gastric organoid-derived monolayers represent different regions of the gastric units

Organoids, as well as organoid-derived 2D monolayers, have been used as model to study *H. pylori* infection by us^21,26^ and others^12,22,27^. Directly comparing 3D versus 2D infection, we find different results regarding the ability of the bacteria to bind to and translocate CagA into the host cell (Supplementary Fig. 1). This prompted us to ask, whether the two systems may be more different than initially thought.

**Fig. 1:**
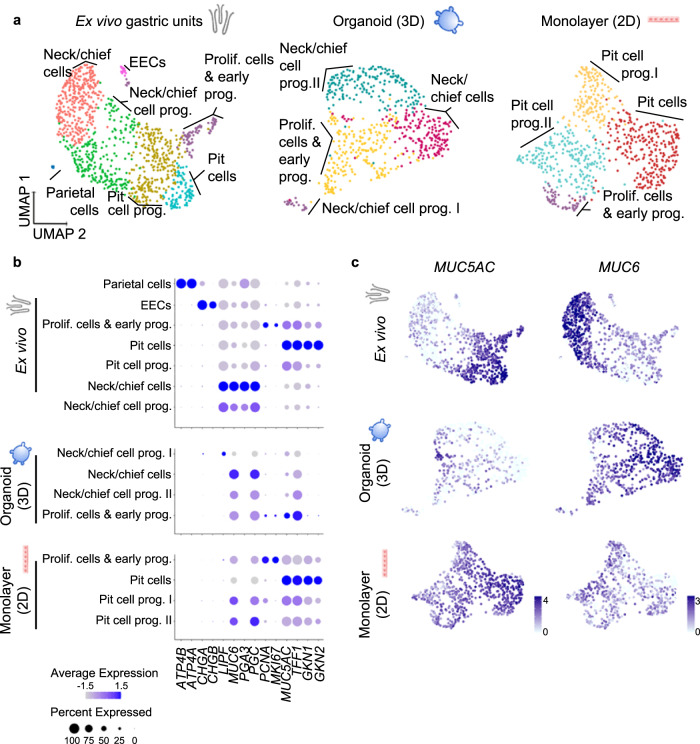
Organoids and organoid-derived monolayers represent different regions of the gastric units. **a** UMAP visualisations of scRNA-seq of ex vivo isolated human gastric units (left, 1022 cells), gastric organoids (centre, 602 cells) and organoid-derived monolayers (right, 696 cells). EEC enteroendocrine cells. Cells are colour-coded according to the annotated clusters. **b** Dot plot showing the expression of cell type-specific marker genes within the clusters as identified in panel (**a**). **c** Expression of the pit cell marker *MUC5AC* and neck cell marker *MUC6* colour-coded and projected on top of the UMAP visualisations as in panel **a** (data was normalized using ‘LogNormalized’ Seurat method for the expression of the gene within the dataset).

Therefore, we aimed to characterize the cell identities present in gastric organoids and 2D monolayers and compare them to freshly ex vivo isolated epithelium. To this end, we sorted freshly isolated human gastric epithelial cells based on the epithelial cell adhesion molecule (EpCAM), 3D organoids and 2D monolayers, and profiled 1241 cells from ex vivo gastric units, 713 cells from organoids and 868 cells from 2D monolayers. Of these, 1022, 602, and 696 cells, respectively, passed quality control. Unsupervised clustering using Seurat software package^28^ identified 7 clusters in the ex vivo gastric units (Fig. 1a, left panel) and cluster identities were assigned based on their expression levels of cell-type-specific markers (Fig. 1b; Supplementary Data 1 and 2, Supplementary Fig. 2).

Comparing the in vitro cultured cells of organoids and 2D monolayers to the ex vivo epithelial cells, similar cell types were identified with the exception of enteroendocrine and parietal cells, which are rare or absent in organoids^21^ (Fig. 1a, middle and right panel). Taking a closer look, however, the two cultures were differentiated into opposing directions, which was apparent in the analysis of the main markers *MUC5AC* and *MUC6* (Fig. 1c).

In the 3D organoids, only few cells in one cluster expressed some levels of *MUC5AC*, but simultaneously *PGC, MUC6* and proliferation markers *PCNA* and *MKI67*, indicating that they were proliferating cells and early progenitor cells (Fig. 1a, b, Supplementary Data 1, Supplementary Fig. 2). Cells from the other three clusters expressed different levels of neck and chief cells markers such as *MUC6* and *PGC* and were annotated as neck/chief-cells and neck/chief progenitors I and II. Gastrokine 1 (*GKN1*) and 2 (*GKN2*) were only expressed at very low levels by a few cells in the organoid sample, indicating that organoids do not contain any mature pit cells (Fig. 1b).

The analysis of 2D monolayers gave the mirror image to the organoids (Fig. 1b, c). *MUC6* and *PGC* were expressed by one neck/chief progenitor cluster, but no mature neck/chief cells were observed in the 2D monolayers. Instead, and opposite to the organoid, the biggest cluster of cells was composed of pit cells expressing high levels of *MUC5AC*, *TFF1*, *GKN1* and *GKN2* comparable to the mature differentiated pit cells of the ex vivo gastric units (Fig. 1a, b, Supplementary Data 1, Supplementary Fig. 2). qRT-PCR analysis corroborated this data (Supplementary Fig. 2b).

These data suggested that gastric organoids rather resemble the gland region, while 2D monolayers resemble the pit region. We hypothesized that the initially observed different capacities of *H. pylori* to adhere to host cells may be rooted in the host cell identities present in the respective system and a preference of the bacteria to adhere to a specific target cell.

### scRNA-seq identifies *H. pylori* tropism

**Fig. 2:**
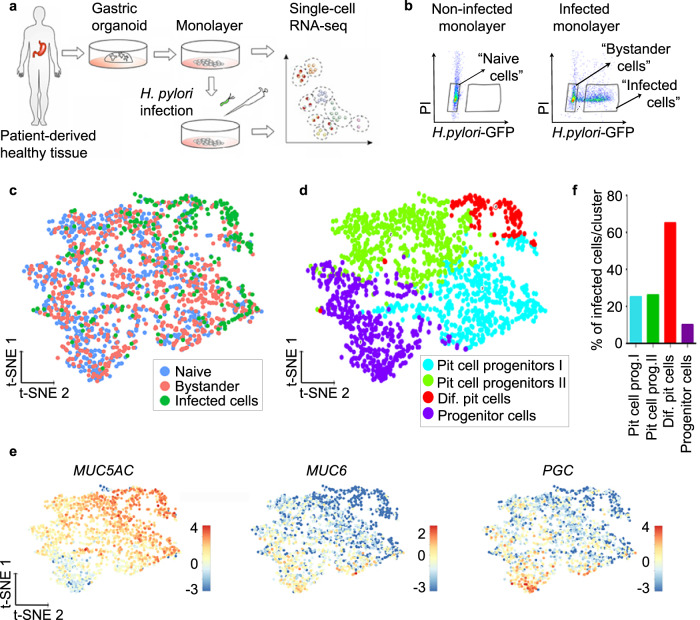
scRNA-seq reveals that *H. pylori* binds to a subpopulation of pit cells. **a** Scheme of the experimental setup. Gastric organoids were generated from gastric healthy tissue and used to obtain 2D monolayers. Monolayers were infected with GFP-expressing *H. pylori* (MOI 1) for 6 h and subjected to scRNA-seq. **b** Representative flow cytometry dot plots of naïve and infected monolayers. Cells were gated as indicated based on GFP signal (*H. pylori*) and propidium iodide (PI) and subjected to scRNA-seq. **c** Single-cell transcriptomes from the different gates (panel **b**) were integrated and projected using a t-distributed stochastic neighbour embedding (t-SNE) 2D projection. The sample origin is colour-coded. **d** Using known gastric marker genes for the specific gastric cell types, cell identities were assigned. **e** Expression of known markers specific for pit cells (*MUC5AC)*, neck cells (*MUC6*) and chief cells (*PGC*) colour-coded and projected on top of the t-SNE displayed in panels (**c**) and (**d**). **f** Percentage of *H. pylori*-infected cells per cluster as identified in panel (**d**).

To identify a possible target cell of *H. pylori*, we performed scRNA-seq of infected cells. For this, cells from human gastric organoids were seeded to form monolayers and infected with GFP-expressing *H. pylori* at a multiplicity of infection (MOI) of 1 or left untreated (Fig. 2a). After 6 h, cells were sorted based on GFP signal, resulting in the 3 samples “naïve” (=untreated), “infected” (GFP-positive) and “bystander” with bystander cells defined as GFP negative cells sorted from an infected sample (Fig. 2b). At this MOI, an infected sample contained usually 40% of infected and 60% of bystander cells. In total, we profiled 2170 cells (868 naïve, 870 bystander and 432 infected). Of these, 1727 cells (713 naïve, 742 bystanders and 272 infected) passed the strict quality-control thresholds (see the “Methods” section for details). The generated data from the three samples was combined and analysed using the Rare Cell Type Identification (RaceID) algorithm^29^. *T*-distributed stochastic neighbour embedding (*t*-SNE) projection showed that *H. pylori*-infected cells clustered together, while bystander cells were interspersed between the naïve cells, indicating that many of the infected cells, but not the bystander cells, differed from the naïve cells based on their transcriptome (Fig. 2c). A total of four clusters were identified based on their transcriptomic profile and annotated based on cell type-specific marker expression (Fig. 2d, e, Supplementary Fig. 3, Supplementary Data 3).

Two clusters were composed of pit cell progenitors (clusters 1 and 2), and 1 cluster was composed of progenitor cells (cluster 3). While the smallest cluster, cluster 4, containing 8% of the total number of cells in the sample, represented the highest differentiated pit cells (Fig. 2d, e, Supplementary Fig. 3, Supplementary Data 4), it also contained the highest percentage of infected cells (65%). In contrast, in clusters 1 and 2, both representing pit cell progenitors, 25% and 26% of cells were infected, respectively. In cluster 3, representing the least differentiated cells, only 10% of cells were infected (Fig. 2f, Supplementary Data 4). Together, the data indicate that *H. pylori* is not restricted to, but preferentially binds to a specific subpopulation of pit cells.

### The *H. pylori* preferential target cell is the most differentiated pit cell expressing high levels of *GKN1, GKN2, CEACAM5, PHGR1* and PSCA

To further characterize this target cell population of *H. pylori*, we focused on the specific transcriptional profile of these cells.

**Fig. 3:**
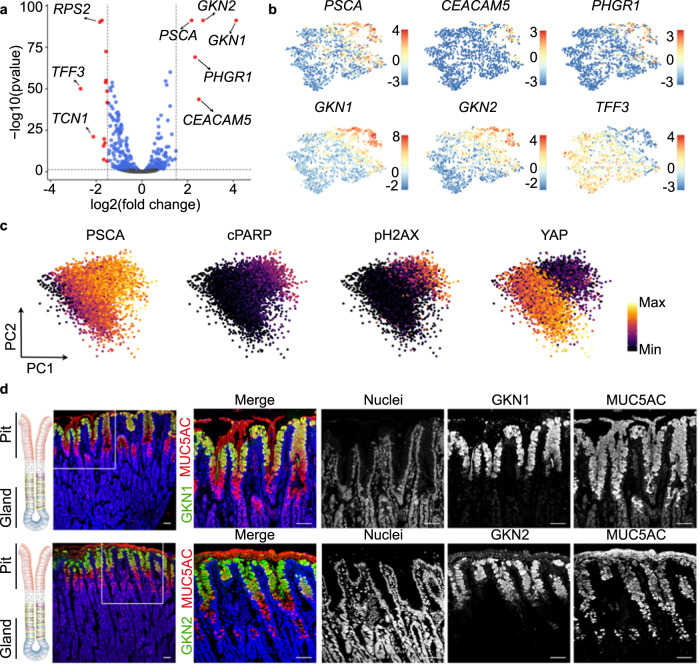
*H. pylori* target population is characterized by a high level of cell differentiation. **a** Volcano plot showing log-fold change and −log (*p*-value) for the comparison between cluster 4 (*H. pylori*-infected enriched cluster) and the rest of the clusters (clusters 1, 2 and 3). Red dots denote DEG with *p*-value ≤ 0.05 and ≥1.5 log2 fold change. Blue dots denote DEG with *p*-value ≤ 0.05 and ≤1.5 log2 fold change. **b** Expression of several DEG shown in panel (**a**). Expression is colour-coded and projected on the t-SNE as in Fig. 2c. **c** Protein levels of PSCA, cleaved PARP, phosphorylated H2AX and YAP in 2D naïve monolayers measured by mass spectrometry (CyTOF). Every dot represents an individual cell and colours denote the intensity of the signal. **d** Confocal microscopic image of human stomach mucosa co-stained for MUC5AC (red), GKN1 (green, upper panel) and GKN2 (green, lower panel) using immunofluorescence. Nuclei were counterstained using Hoechst 33342 (blue). Scale bar: 50 µm. Data in panel **c** is representative results of organoid lines derived from 3 donors and 1 experiment. Data in panel **d** is representative of results from 2 donors and 1 experiment.

Comparison of the preferential target cell (cluster 4) with the less differentiated cells (clusters 1–3) identified 19 differentially expressed genes (DEG), 5 genes with higher and 14 genes with lower expression in the differentiated pit cells (log2 ≥ 1.5, *p*-value ≤ 0.05) (Fig. 3a, Supplementary Data 5).

We focused on 5 highly expressed genes: *GKN1*, *GKN2*, *CEACAM5*, prostate stem cell antigen (*PSCA*) and proline, histidine and glycine-rich 1 (*PHGR1*; Fig. 3a). Similar to *MUC5AC* (Fig. 2f), the expression of these genes followed a gradient with the highest expression level in cluster 4 (Fig. 3b). Mass cytometry indicated that cells with the highest expression for PSCA were also positive for apoptosis markers (phosphorylated H2A histone family member X, pH2AX; cleaved Poly ADP-ribose polymerase, cPARP) and negative for YAP (Fig. 3c, Supplementary Fig. 4). Both suggested that the gradient indicates cellular differentiation and that the cluster 4 may constitute a highly differentiated pit cell population. To verify this hypothesis, we analysed the protein expression in histology. In agreement with previous studies^30,31^, GKN1 and GKN2 marked a fraction of MUC5AC-positive pit cells localized at the opening of the pit (Fig. 3d). Classical studies using autoradiography have identified these cells as the most matured pit cells^32^. Data from the Human Protein Atlas^33^ showed a similar localization for PHGR1, PSCA, and CEACAM5 (Supplementary Fig. 5). qRT-PCR analysis of these markers after infection demonstrated that their presence in infected cells was not due to the upregulation of the gene transcripts upon infection (Supplementary Fig. 6a). No bacterial binding preference was observed when using higher MOIs for infection. At an MOI of 100, *H. pylori* attached to all cells independent of the marker gene expression (Supplementary Fig. 6b, c).

These results indicate that *H. pylori* preferentially binds to a subpopulation of highly differentiated pit cells, characterized by expression of *GKN1*, *GKN2*, *PHGR1, PSCA* and *CEACAM5*, located in the upper pit region.

### Directed differentiation of 2D monolayers

**Fig. 4:**
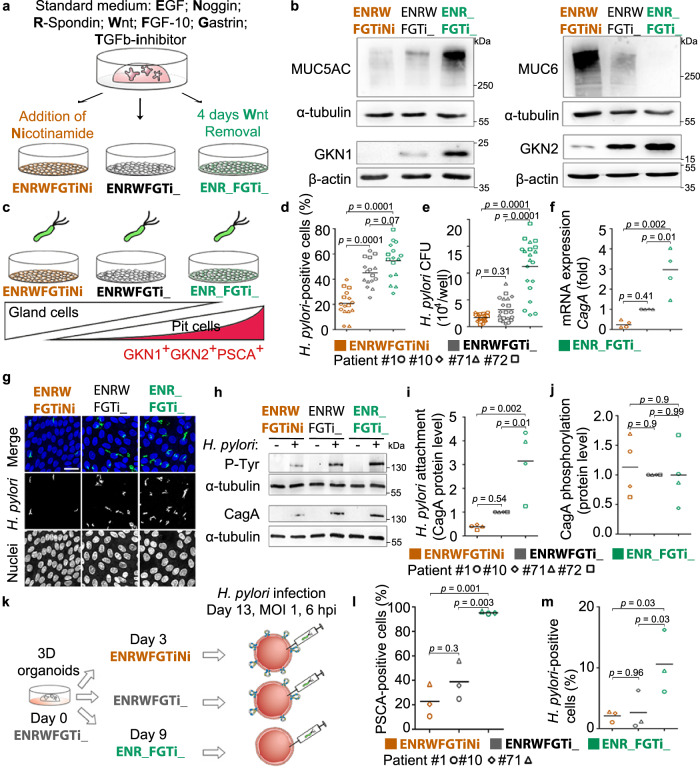
Increased number of differentiated pit cells leads to higher *H. pylori* adhesion. **a** Scheme of the directed differentiation setup. **b** Western blot analysis of pit markers (*MUC5AC*, *GKN1*, and *GKN2*) and neck markers (*MUC6*) in 2D monolayers upon differentiation according to the scheme in (**a**). **c** Scheme of the experimental setup. Differentiated monolayers were infected with a *H. pylori* strain expressing GFP at a MOI of 1 for 6 h. **d–f** Quantification of adhered bacteria to 2D monolayer cells by (**d**) flow cytometry, (**e**) CFU assay and (**f**) qRT-PCR of *CagA* expression (fold over host *GAPDH*; **f**). Results are normalized against standard media (ENRWFGTi_, grey symbols). **g** Confocal images of differentiated gastric 2D monolayers infected with GFP-expressing *H. pylori*. Nuclei were counterstained with Hoechst 33342. Scale bar: 25 µm. **h** Western blot analysis of phosphorylated CagA (P-Tyr) in *H. pylori*-infected 2D monolayers. **i** and **j** Quantification of bacterial attachment (CagA total protein over host alpha-tubulin; **i** and CagA translocation (P-Tyr over CagA; **j**) by western blot. **k** Scheme of the experimental setup. Differentiated 3D organoids were microinjected with *H. pylori* (MOI 1 for 6 h). **l** Quantification of PSCA-positive cells in 3D organoids measured by flow cytometry. **m** Quantification of adhered bacteria to 3D organoid cells by flow cytometry. Data from organoid lines derived from individual donors are shown as symbols (patient #1-circle, #10-diamond, #71-triangle, and #72-square), with mean values with horizontal lines. Data in panel **b**, **d**–**j** are representative of or show data of organoid lines from 4 donors, **l** and **m** of 3 donors, and 1 (**b**, **f**, **i** and **j**), 3 (**g**, **h**, **l**, **m**), 4 (**d**) or 5 (**e**) independent experiments. Statistical analysis of data in panels **d**–**f**, **i**, **j** and **l**, **m** was performed using one-way ANOVA with Tukey’s multiple comparisons post-hoc test. Source data are provided as a Source Data file.

To verify the preferential binding for *H. pylori*, we aimed to control the abundance of the differentiated pit cells in the 2D monolayers, using directed differentiation as published before^21^. For this, standard growth conditions (EGF, Noggin, R-Spondin, Wnt, FGF-10, Gastrin and TGFβ inhibitor or in short ENRWFGTi) are modified to direct the differentiation of the cells (Fig. 4a)^21^. Wnt withdrawal led to differentiation towards the *H. pylori* target cell, as indicated by high expression of *MUC5AC, GKN1, GKN2* and *PSCA* on RNA and protein levels (Fig. 4b, Supplementary Fig. 7a), while the addition of nicotinamide led to differentiation towards the gland cell identities as indicated by increased expression of *MUC6* and *PGC* (Fig. 4b, Supplementary Fig. 7a). Flow cytometric analysis demonstrated that Wnt withdrawal induced an increase in size (median FSC 6.24 × 10^6^ in ENR_FGTi_ compared to 5.10 × 10^6^ in ENRWFGTiNi; Supplementary Fig. 7b) and granularity (median SSC 1.10 × 10^6^ in ENR_FGTi_ compared to 7.68 × 10^5^ in ENRWFGTiNi; Supplementary Fig. 7c), giving the impression of differentiation towards a large and granular cell, matching the expectation for highly differentiated pit cells. Analysis of PSCA staining in these conditions showed the differentiation to a PSCA^+^SSC^high^ population (37.2 ± 9.1 % of gated cells in ENR_FGTi_ compared to 10.4 ± 4.8% in ENRWFGTiNi; Supplementary Fig. 7d, e). Moreover, during the differentiation towards the pit cell lineage, higher production of mucus was observed in the culture medium compared to the control (ENRWFGTi_, Supplementary Movie 1). These results show that three types of 2D monolayers can be generated that differ in their content of pit- or gland-type cells by modifying the composition of the culture media.

### *H. pylori* adherence and CagA translocation match the presence of differentiated pit cells in differentiated monolayers

Using the three different growth conditions, we then analysed, whether the absence or presence of the target cell would affect bacterial binding and CagA translocation. For this, 2D monolayers were infected with *H. pylori* MOI 1 (Fig. 4c). Flow cytometric analysis showed 20.7%, 45% or 54.5% of *H. pylori*-positive cells in the gland cell differentiation, standard, or pit cell differentiation condition, respectively (Fig. 4d), indicating that bacterial binding increases with the presence of mature differentiated pit cells. This was matched by 1.7, 3.2 and 11.2 × 10^4^ colony forming units (CFU), respectively, as measured by classical attachment assay (Fig. 4e). Also, expression levels of *CagA* indicated the similar distribution of bacterial RNA in the infected samples (Fig. 4f) and confocal imaging of the infection confirmed the higher bacterial adherence to the pit cell differentiation condition (Fig. 4g). Western blot also showed about 3-fold higher level of CagA protein in the pit cell differentiation condition. Western blot for phosphotyrosine showed that the bound bacteria also actively injected CagA into the host cells (Fig. 4h–j). To test whether other factors specific for 2D or 3D would influence binding, we used directed differentiation in 3D, which also yielded the differentiated pit cells. Flow cytometry analysis of organoids microinjected with bacteria showed that *H. pylori* attachment is similarly correlated to PSCA expression in 3D as it is in 2D (Fig. 4k–m).

Together, these results indicate that *H. pylori* preferentially binds to differentiated pit cells and that this differentiation can be directed to increase bacterial adhesion both in 2D and 3D.

### Binding of *H. pylori* to differentiated pit cells is independent of MUC5AC and PSCA

We hypothesized that specific proteins might mediate the preference to the target cell. Candidate proteins were MUC5AC because previous studies have demonstrated the binding of *H. pylori* to Lewis B antigens of MUC5AC^6,34^ and PSCA because it is the highest expressed surface antigen-specific for this cell type in our analysis. To analyse the importance of MUC5AC and PSCA, we generated CRISPR/Cas9-mediated knockouts in human gastric organoids. Knockout was confirmed in DNA and protein analysis (Supplementary Fig. 8), but bacterial binding indicated that neither MUC5AC nor PSCA are required for *H. pylori* adhesion to the 2D monolayers (Supplementary Fig. 8).

### Preference for pit cell depends on urea chemotaxis and cell size

We then analysed the possible role of secreted factors. To test whether gland cells would secrete bactericidal proteins to repel or kill bacteria, we incubated bacteria with supernatants from the differentiated cells. The supernatants did not have an effect, although infection did lead to the upregulation of some known antimicrobials (Supplementary Fig. 9).

**Fig. 5:**
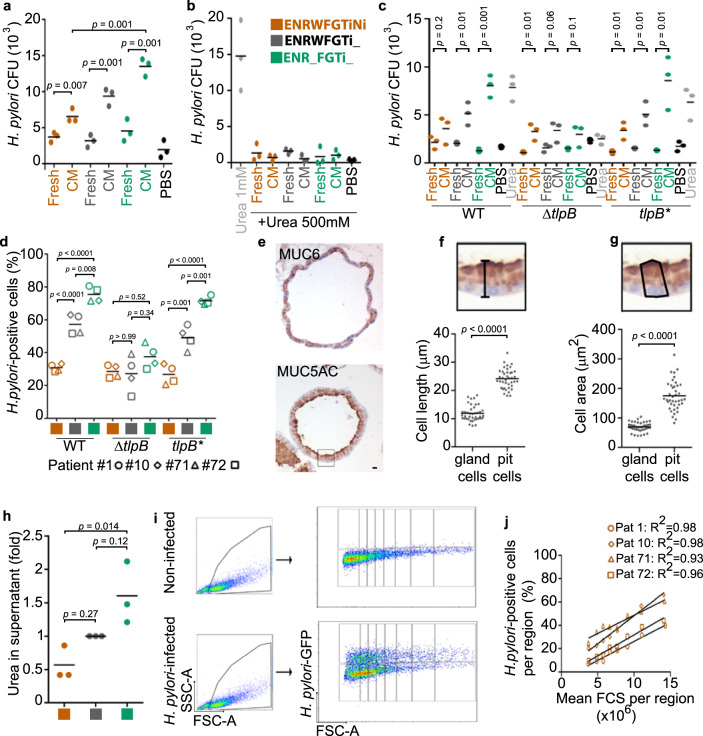
*H. pylori* preference for pit cells depends on chemotaxis to urea scaling with size. **a–c** Quantification of wild-type (wt, strain P12) bacterial chemotaxis towards conditioned media (**a**) or conditioned media plus 500 mM urea (**b**). Chemotaxis by *H. pylori* strain G27 WT, Δ*tlpB* and tlpB* (**c**). **d** Quantification of adhered *H. pylori* strains G27 WT, Δ*tlpB* and tlpB* to organoid-derived monolayers by flow cytometry. **e** Representative immunohistochemistry images of a 3D organoid stained for MUC5AC and MUC6. Scale bar: 10 µm. **f** and **g** Quantification of cell length (**f**) and cell area (**g**) of MUC5AC-(pit cells) and MUC6-positive cells (gland cells) in 3D organoids. **h** Quantification of urea concentration in organoid-derived monolayer conditioned media. Fold over control differentiation media (grey, ENRWFGTi_). **i** Representative flow cytometry analysis. **j** Quantification of *H. pylori*-positive cells in the FCS regions shown in (**i**). Data are shown as individual dots (panels a-c, and h, filled dot, pool of 4 organoid lines, 3 independent experiments; in panels **d** and **j**, symbols represent organoid lines derived from patient #1-circle, #10-diamond, #71-triangle, and #72-square; 3 independent experiments), mean values with horizontal lines (panel **d**). Data in panel **e** is representative of 2 organoid lines and 1 experiment. Statistical analysis of data in panels **a**–**c** was performed using multiple *t*-test corrected for multiple comparisons with the Holm–Sidak method; in panels **d** and **h**, one-way ANOVA with Tukey’s multiple comparisons post-hoc test.; and in panels **f**, **g**, two-tailed Student’s *t*-test.

To test whether pit cells would attract bacteria, we employed a classical chemotaxis assay^35^. All cell-conditioned media attracted bacteria, but conditioned medium from pit cells attracted about twice as many bacteria as conditioned medium from gland cells (Fig. 5a). Urea is a major chemoattractant for *H. pylori*^36^. To test whether the here observed chemotaxis was due to urea, we added 500 mM of urea, which has been shown to abrogate *H. pylori* chemotactic response to urea^36^. Indeed, the high urea abrogated the response to the pit cell-conditioned media (Fig. 5b). Deletion of *TlpB*, the *H. pylori* gene for the chemoreceptor that recognizes urea^36^ abrogates the preference for the pit cells (Fig. 5c). TlpB mutant bacteria also did not prefer pit cells in infections (Fig. 5d).

Urea is a byproduct of amino acid catabolism. Because larger cells produce more protein^37^, it is likely that large cells may secrete more urea than small cells. As mentioned above, already flow cytometry analysis had shown that cells in the ENR_FRTi_ condition (pit cells) are larger than cells in ENRWFGTiNi condition (gland cells) (Supplementary Fig. 7b–e). This is also visible in the histological staining of organoids (Fig. 5e–g). Matching cells in the ENR_FRTi_ condition (pit cells) produce about 3 times higher concentrations of urea than cells in the ENRWFGTiNi condition (gland cells) (Fig. 5h). If urea secretion scaling with size would be a major determinant of *H. pylori* preference, this would also be visible in all differentiated cells (irrespective whether these are pit or gland cells). Indeed, flow cytometry analysis showed that percentages of *H. pylori*-infected cells correlate to the cell size also in the absence of pit cells (ENRWFGNiTi) (Fig. 5i, j).

We conclude that urea chemotaxis leads to *H. pylori* preferential binding to large cells and that the binding preference to differentiated pit cells is rooted in the cell size.

### *H. pylori* localises in close proximity to highly differentiated pit cells in vivo

**Fig. 6:**
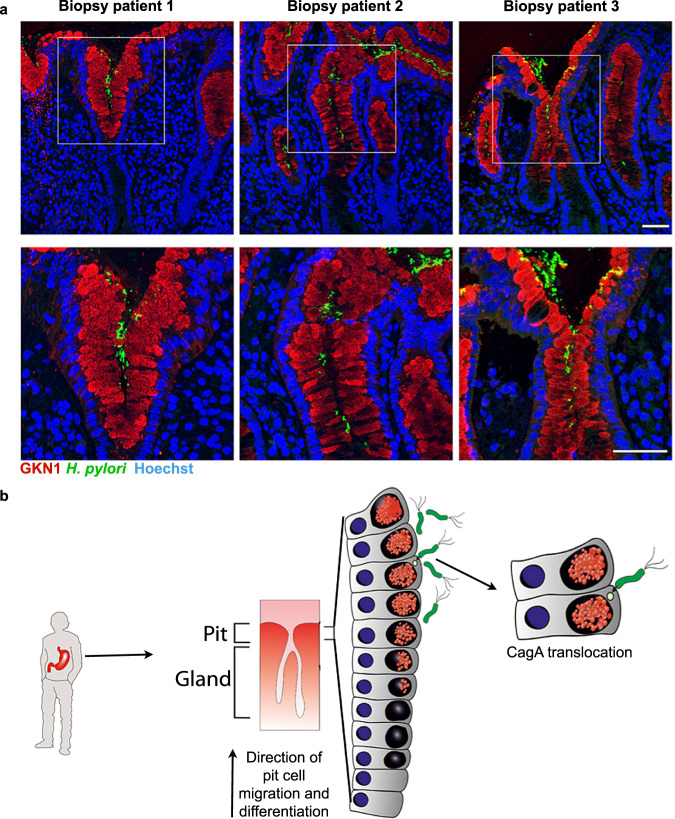
*H. pylori* colocalizes with differentiated pit cells in vivo. **a** Representative confocal microscopy images of *H. pylori*-positive tissue sections co-stained for GKN1 (red) and *H. pylori* (green). Nuclei were counterstained using Hoechst 33342 (blue). Scale bar: 50 µm. Data shown are representative images of three patients and one experiment each. **b** Model: *H. pylori* preferentially binds to and translocates CagA into highly differentiated pit cells located at the opening of the gastric units.

Having observed preferential binding of *H. pylori* to a subpopulation of highly differentiated pit cells in vitro, we examined whether this preference could be observed in vivo. For this, we used *H. pylori*-positive gastric biopsies from donors that underwent endoscopy diagnosis. Immunofluorescence microscopy showed that *H. pylori* was predominantly found next to cells expressing GKN1 at the opening of the gastric pits (Fig. 6a).

Overall, these observations demonstrate that *H. pylori* preferentially binds to highly differentiated pit cells, located in the upper area of the gastric pits, where it can translocate its CagA virulence factor (Fig. 6b).

## Discussion

For *H. pylori*, bacterial adhesion is not only an important step for colonization in the human stomach^10^, but also a prerequisite for CagA translocation^7,8,10^. In this study, we demonstrate that *H. pylori* preferentially binds to a highly differentiated subpopulation of gastric pit cells. This is mirrored by high bacterial density in the pit region in patient tissue.

Already classical studies by Warren and Marshall reported that in human biopsy material, the majority of *H. pylori* is found close to the pit region^38^. This preferential location was underlined by quantification: In human biopsy material, less than 1% of bacteria are found associated with MUC6-producing gland cells^39^. Studies in mouse sections find more frequent colonization of the deep glands ^4,40^. A direct quantitative comparison between bacterial location in mouse and human infection is lacking, but the current data suggests possible differences between hosts.

Human organoids and their derived monolayers are emerging additions to the spectrum of model systems in infection biology and allow the study of human-specific pathogens with primary human epithelium. Here, a comparison of ex vivo sorted gastric epithelial cells with cells from 3D organoids and organoid-derived 2D monolayers using single-cell sequencing demonstrated that the two models represent different parts of the gastric unit: Under standard growth conditions, 2D monolayers harbour mostly cells from the pit region and their progenitors, while 3D organoids rather harbour the glandular progenitors. Both models are important for the understanding of *H. pylori* infection, and both can be directed towards different cell fates. Because organoids and their derived monolayers are now becoming a key model in a wide range of bacterial and viral infections^12,21,22,41–48^, the cell identities present should be taken into consideration when choosing the experimental system and interpreting results.

In this study, we have characterized the highly differentiated pit cell as a preferential target cell for *H. pylori* binding. This subset of pit cells is characterized by expression of high levels of *MUC5AC, GKN1, GKN2, CEACAM5, PSCA* and *PHGR1*. MUC5AC contains Lewis B antigens, which are known to be bound by *H. pylori* adhesins. PSCA is a membrane-anchored glycoprotein expressed in several organs including the stomach. Its function has not been elucidated yet, but genetic variants of this gene have been associated with gastric cancer^49^. However, in our experiments, MUC5AC or PSCA expression was dispensable for the binding. CEACAM5 is a receptor for HopQ, an *H. pylori* adhesin^7,8^. In our sample, *CEACAM5* transcript was only detected in a fraction of the target cell population and thus it is likely that this is not the only factor important for bacterial binding in our setting. Instead, we found that chemoattraction to the host cell metabolite urea underlies the preferential binding to differentiated pit cells. Urea attraction has been described early using capillary tube assays^50,51^. Recent work has identified that *H. pylori* directly senses urea via the chemoreceptor TlpB^36^. TlpB has an unusually high affinity for urea and *H. pylori* can detect concentrations of as low as 50 nM. Simultaneous degradation of urea by *H. pylori*-secreted urease prevents the receptor from being saturated. This highly sensitive urea-sensing by TlpB is important for the long-term colonization of the stomach^36^. Interestingly, *TlpB* gene expression is regulated via targeting of a variable G-repeat in its sequence by the small regulatory RNA RepG^52^, which was also recently shown to regulate HP0102, a gene involved in LPS biosynthesis^53^.

The importance of cell size, for example in aging and stem cell capacity, is only beginning to be discussed^54,55^. Here we show that the urea sensing of *H. pylori* leads to a bacterial preference for larger cells. Notably, large size characterizes senescent cells^56,57^. Our size measurements in organoids correspond well to measurements in human organoid-derived monolayers^22^ and mouse tissue, with heights for gland cells from 13 µm to 19 µm, and heights for pit cells of 20–45 µm, with the length of the pit cells increasing with differentiation^19,58^. Notably, pit cells are the cells with the highest turnover and are being replaced about every 3 days^59^. In addition, contrary to gland cells, pit cells are refractory to mounting an inflammatory response to infection with *H. pylori*^21^. Thus, with this very sensitive chemotaxis towards urea, evolution has shaped a fine-tuned system, in which bacteria target those cells that are not only mounting the lowest immune response but, are also replaced most frequently in the epithelium and thus most dispensable for the tissue.

## Methods

### Gastric organoid generation and organoid-derived monolayer seeding

Human gastric tissue was obtained from four donors (age range 32–82 years) that underwent partial gastrectomy at the University Hospital of Würzburg. This study was approved by the ethical committee of the University of Würzburg (Approval 37/16) and informed consent was obtained from all the donors.

Generation and culture of gastric organoids were performed as described before^21,60^. For propagation, organoids were split every 12 days at a ratio of 1:10 using mechanical splitting. Culture medium was replaced every 2–3 days. Propagation culture medium contained: Advance Dulbecco’s modified Eagle medium/F12 (12634028, Thermo Fischer Scientific) supplemented with 10 mmol l^−1^ HEPES (15630056, Thermo Fischer Scientific), 1x GlutaMAX (35050-038, Thermo Fischer Scientific), 1xB27 (12587010, Thermo Fischer Scientific), N-Acetylcysteine 1 mM (A9165-25G, Sigma-Aldrich), Noggin conditioned medium 10%, R-spondin1 conditioned medium 10%, Wnt conditioned medium 50%, EGF 50 ng ml^−1^ (AF-100-15, Peprotech), FGF10 200 ng ml^−1^ (100-26, Peprotech), Gastrin 1 nM (3006, Tocris), TGFβi 2 µM (A-83-01, Tocris), Primocin 100 ng ml^−1^ (Ant-pm-1, Invivogen). The propagation culture medium was named ENRWFGTi_. After seeding RHOKi 10 µM (Y-27632, Sigma-Aldrich) was added.

Gastric organoid-derived monolayers were generated from 8-day-old organoids. Organoids were collected and mechanically disrupted. Organoid fragments were washed with Advanced DMEM/F12 containing GlutaMAX 1x (35050-038, Thermo Fischer Scientific), 10 mM HEPES (15630056, Thermo Fischer Scientific) and centrifuged at 400×*g* for 5 min. Organoid fragments were resuspended in TrypLE Express (12605028, Gibco) and incubated at 37 °C for 10 min. Single-cell suspension was washed and centrifuged at 400 × *g* for 5 min. Single cells were counted and seeded at 15,000 cells/well in 48-well plate (833923, Sarstedt) using the propagation culture media (ENRWFGTi_). After seeding RHOKi 10 µM (Y-27632, Sigma-Aldrich) was added. Monolayers were allowed to grow and differentiate for 14 days to reach 90–100% confluency. Six days prior to infection, Primocin was exchanged for *H. pylori* compatible antibiotics (vancomycin, nystatin and trimethoprim, concentration as described for the bacterial cultures).

To direct the culture toward the gland cell differentiation, nicotinamide 10 nM (N0636, Sigma-Aldrich) was added to the culture media on day 4 (for organoid-derived monolayers) or day 3 (for 3D organoids; ENRWFGTiNi). To induce differentiation into pit cells, Wnt was removed from the culture medium on day 10 (for organoid-derived monolayers) or day 9 (for 3D organoids; ENR_FGTi_).

### Bacterial culture and infection

The clinical isolates *H. pylori* strain P12 and its derivate expressing GFP constitutively from a chromosomal locus were previously described^61^ and it was a kind gift of Thomas F. Meyer. The *H. pylori* isolate strain G27, Δ*tlpB* and *tlpB* complemented strain (tlpB*) were previously described^36^ and a kind gift from Manuel Amieva. We also received G27 and *tlpB* mutant from Cynthia Sharma^52^ and this showed the same results. *H. pylori* was grown on GC agar plates (CM0367B, Oxoid) containing 10% of heat-inactivated horse serum (S0900-500, Biowest) and supplemented with nystatin 0.2 µg ml^−1^ (N3503, Sigma-Aldrich), trimethoprim 0,25 µg ml^−1^ (T7883, Sigma-Aldrich), vancomycin 1 µg ml^−1^ (0242.3, Carl Roth) and kanamycin 8 μg ml^−1^ (C0378, Sigma-Aldrich). All bacterial cultures were incubated under microaerobic conditions (85% N_2_, 10% CO_2_, 5% O_2_) at 37 °C and in the case of liquid culture with orbital shaking (140 rpm).

For infection, bacteria were grown on plates for 72 h, before passaging to a fresh plate and let to grow for an additional 24 h. A day before infection, scrapes from agar were used to inoculate 10 ml of brain heart infusion (BHI) broth supplemented with 10% heat-inactivated FBS (S0615/1109D, Merck Millipore) and antibiotics for 12 h. This culture was used to set up an overnight culture (14 h) for the infection. For this, the 12 h *H. pylori* culture was diluted to an OD_600_ of 0.05 in BHI broth and grown at 37 °C under microaerobic conditions (85% N_2_, 10% CO_2_, 5% O_2_) with shaking until OD_600_ 0.5 (14 h). Bacteria were harvested by centrifugation and resuspended in the organoid culture medium. Bacteria were then added apically onto the organoid-derived monolayers with an MOI of 1 for 6 h unless otherwise indicated. After 6 h incubation, unbound bacteria were removed by 3 washes with PBS and cells were collected and further processed for western blot, flow cytometry, RNA isolation, microscopy, or scRNA-seq as described below. To quantify bacterial adhesion of the *H. pylori* strains G27MA, Δ*tlpB* and tlpB* to the organoid-derived monolayers by flow cytometry, bacteria were fluorescently labelled prior infection. Briefly, overnight bacterial cultures were obtained as described above, harvested by centrifugation, washed once with PBS and incubated with 1 ml of PBS containing 1 µM eFluor 670 (65-0840-85, Invitrogen™) for 10 min at 37 °C. Then, bacteria were centrifuged and incubated with BHI medium for 2 min at RT, washed twice with PBS and resuspended in organoid culture medium.

For infection of 3D human gastric organoids, organoids were microinjected with *H. pylori* as previously described^21^. Briefly, organoids were seeded in 50ul of Matrigel in a 4 well multidish (144444, Thermo Fisher Scientific) and on day 13, organoids were microinjected with an approximate MOI of 1. To achieve the final MOI, bacteria were resuspended in Advanced DMEM F12 at a density of 2 × 10^7^ bacteria ml^−1^ (MOI 1) and organoids were injected with approximately 0.2 µl bacterial suspension using a micromanipulator and microinjector (M-152 and IM-5B, Narishige) under a stereomicroscope (Leica MZ75) inside a biosafety cabinet.

### Flow cytometry and CFU analysis

After infection, cells were washed three times with PBS, incubated with TrypLE Express (12605028, Gibco) for 10 min, and washed twice with FACS buffer (PBS + 10% FBS). Cells were incubated with anti-PSCA Alexa 647 conjugated antibodies (1:50, sc-80654, Santa Cruz Biotechnologies) for 30 min at 4 °C in dark. Cells were then washed twice and resuspended in a solution of 50 ng ml^−1^ of propidium iodide (51-6621E, BD biosciences) in FACS buffer. Single cells were gated by using forward scatter area versus forward scatter peak linear. Dead cells were excluded by propidium iodide staining. PSCA and GFP (*H. pylori* P12-GFP) signals were measured in 10,000 cells. Cells were analysed using the Accuri^TM^ C6 (BD Biosciences) and data were analysed with the Accuri^TM^ C6 software (BD Biosciences) and FlowJo^TM^ software package (BD Biosciences).

To quantify bound bacteria by CFU assay, after infection, cells were washed three times with PBS and lysed with 0.1% saponin (A4518-0100, AppliChem) in PBS. Cell lysates were serially diluted, plated in GC agar plates and left to grow for 4 days.

### RNA isolation and qRT-PCR

Isolated gastric units, organoids or cell monolayers were washed with PBS and harvested in RLT buffer (79216, Qiagen). Total RNA was extracted using the RNeasy Mini Kit (74106, Qiagen) according to the manufacturer’s instructions. RNA was then reverse-transcribed using hexameric random primers (48190011, Life Technologies) and M-MuLV reverse transcriptase (M0253, New England Biolabs) according to the manufacturer’s instructions. Quantitative real-time PCR analysis was performed according to the manufacturer’s instructions, in a CFX96 Touch Real-Time PCR detection system using Bio-Rad CFX Manager software (BioRad). The 10 μl PCR reaction included 1 μl of 1:5 diluted cDNA as template, 5 μl of SsoAdvanced Universal SYBR Green Supermix (172-5270, BioRad) and 1 μl of each primer (10 μM). Primers used in this study were: *MUC5AC* 5′-CTTCTCAACGTTTGACGGGAAGC-3′ and 5′-CTTGATCACCACCACCGTCTG-3′, *MUC6* 5′-GCCCCGGTATCTTCTCTCGG-3′ and 5′-ACACCTGCAGGGTGAGTACG-3′, *PGC* 5′-AGAGCCAGGCCTGCACCAGT-3′ and 5′-GCCCCTGTGGCCTGCAGAAG-3′, *GAPDH* 5′-CTCTCTGCTCCTCCTGTTCGAC-3′ and 5′-TGAGCGATGTGGCTCGGCT-3′, *GKN1* 5′-GGCCTGATGTACTCAGTCAACC-3′ and 5′-TTTAGTTCTCCACCGTGTCTCC-3′, *GKN2* 5′-TGCAGGATCATGCTCTTCTAC-3′ and 5′-TGGTCCATCTTCAGGATAAAG-3′, *PSCA* 5′- TGCTTGCCCTGTTGATGGCAG-3′ and 5′-CCAGAGCAGCAGGCCGAGTGCA-3′, *PHGR1* 5′- CCCTGCTCTGCACTCTCAG-3′ and 5′-CGCAGTGACCTGGAGGAT-3′, *cagA* 5′-TGGTGTGAATGGAACCCTAGT-3′ and 5′-CCCGCTGCTTGCCCTACACC-3′, *ITLN1* 5′-TCTGTTTGGCATCTACCAGAAAT-3′ and 5′-GATGCTGTTTTCTGGGCGTC-3′, *OLFM4* 5′-AGGTTCTGTGTCCCAGTTGT-3′ and 5′-CAAGCGTTCCACTCTGTCCA-3′ *REG3G* 5′-ATGCTGCTTTCCTGCCTCAT-3′ and 5′-GACAGCTGATCCGTGGAGAG-3′, *LCN2* 5′-CCAGGACAACCAATTCCAGG-3′ and 5′-GGCATACATCTTTTGCGGGT-3′, *LL37* 5′-CAAGAAGGACGGGCTGGTGAA-3′ and 5′-CACAACTGATGTCAAAGGAGCC-3′, *HBD1* 5′-TGAGATGGCCTCAGGTGGTAA-3′ and 5′-CACTTGGCCTTCCCTCTGTA-3′, *HBD2* 5′-CCAGCCATCAGCCATGAGGGTCTT-3′ and 5′-CATGTCGCACGTCTCTGATGAGGGAGC-3′. The 2^−ΔΔCt^ method was used to calculate fold changes.

### Immunohistochemistry

Organoids were fixed with 4% formaldehyde overnight at 4 °C. Paraffin sections and immunohistochemistry staining was performed according to a previously published protocol^21^. Briefly, organoids were permeabilized with 0.3% Triton X-100 and blocked with PBS 0.3% Triton X-100, 1% BSA, and 5% goat serum. MUC5AC (1:10, 45M1, Vision biosystems) or MUC6 (1:200, sc-16914, Santa Cruz Biotechnologies) antibodies were used to stain pit cells or gland cells, respectively. Images were taken using a standard light microscope (Nikon Eclipse E600). Cell length and area were quantified using ImageJ by counting 10 cells per organoid from 2 patients.

### Confocal microscopy

To analyse organoid-derived monolayers, singularized cells were seeded on µ-Slide 8 Wells (80826, Ibidi) and expanded for 14 days. After infection, cells were fixed with 4% paraformaldehyde for 20 min at RT, permeabilized with 0.5% Triton X-100 in PBS for 15 min and then blocked with 1% BSA for 1 h. Primary antibodies were diluted in blocking solution and incubated overnight at 4 °C followed by 1 h at RT. Primary antibody anti-MUC5AC was used at 1:100 (MA5-12178, Thermo Fisher Scientific). The secondary antibody goat anti-mouse Alexa Fluor-488 (1:500, A-11001, Thermo Fisher Scientific) was also diluted in a blocking solution and incubated at RT for 1 h. Nuclei were counterstained with Hoechst 33342 (1:5,000; H3570, Life Technologies).

To analyse human gastric tissue, tissue samples from human stomach biopsies were fixed in formalin 10% (09122, Noegen) and embedded in paraffin. Embedded sections (5 μm) were deparaffinized in xylene and then hydrated in graded alcohol. Citrate buffer (10 mM, pH 6) was used for antigen retrieval using a steamer. Then slides were washed in PBS and permeabilized using 0.5% Triton X-100 in PBS for 30 min, followed by blocking with 1% BSA in PBS 0.5% Tween 20 for 30 min. Primary antibodies used for this study were: anti-GKN1 (1:500, HPA047684, Atlas antibodies-for double staining with anti-MUC5AC antibody; 1:50, AF7287, R&D Systems- for double staining with anti-*H. pylori* antibody), anti-GKN2 (1:500, ab188866, Abcam), anti-MUC5AC (1:100, 45M1, Vision Biosystems). Samples were incubated with the primary antibody in a blocking solution overnight at 4 °C followed by an additional hour at RT. Secondary antibodies were incubated for 1 h at RT. The following secondary antibodies were used: donkey anti-mouse Alexa Fluor 594 (1:250, A-21203, Thermo Fischer Scientific), goat anti-rabbit Alexa Fluor 488 (1:500, 4412S, Cell Signaling Technology), donkey anti-sheep Alexa Fluor 594 (1:500, A-11016, Thermo Fischer Scientific). Nuclei were counterstained with Hoechst 33342 (1:5,000; Life Technologies, H3570). Slides were mounted in Mowiol (8138.1, Sigma-Aldrich).

Confocal microscopy images, shown as maximum projected Z-stack images, were acquired with a Leica SP5 laser scanning confocal microscope and LAS AF Lite software (Leica Microsystems).

### Data mining from “The Human Protein Atlas”

IHC images of human stomach tissue samples stained for MUC5AC, GKN1, GKN2, PSCA, PHGR1, and CEACAM5 were obtained by datamining the human protein atlas database (www.proteinatlas.org)^33^.

### scRNA-seq library preparation and sequencing

For scRNA-seq of *H. pylori*-infected 2D monolayers, cells were washed three times with PBS after infection, incubated with TrypLE Express (12605028, Gibco) for 10 min and washed twice with FACS buffer (PBS + 10% FBS) and resuspended in PBS. The cell suspension was then filtered with a 30 µm filter and sorted based on the signal intensity from the FITC-A channel. Mock-infected cells were also sorted. Dead cells marked with propidium-iodide (1:1000, 556463, BD Biosciences) were excluded.

For scRNA-seq of ex vivo isolated epithelium, gastric units were isolated from gastric biopsies as described before for the organoid cultures. Gastric units were then incubated with TrypLE Express (12605028, Gibco) in order to get a single-cell suspension. The cell suspension was then washed with FACS buffer and stained with EpCAM antibody (1:350, sc-25308, Santa Cruz Biotechnologies) for 30 min on ice. After three washes with FACS buffer, cells were filtered as described above and submitted to sorting. Only EpCAM-positive cells were sorted. Dead cells marked with propidium-iodide were excluded.

For scRNA-seq of 3D organoids, the organoids were taken from the Matrigel dome and dissociated mechanically and enzymatically as described above. Single-cell suspensions were filtered with a 30 µm filter. Dead cells marked with propidium-iodide were excluded.

Single cells were sorted by FACSAria III sorter (BD Biosciences) using FACSDiva software (BD Biosciences) and were collected in 1xPBS containing 0.04% w/v BSA (400 μg ml^−1^) at a concentration of 200-400 cells μl^−1^. Chromium™ Controller was used for partitioning single cells into nanoliter-scale Gel Bead-In-EMulsions (GEMs) and Single Cell 3’ reagent kit v2 for reverse transcription, cDNA amplification and library construction (10× Genomics). The detailed protocol was provided by 10× Genomics. SimpliAmp Thermal Cycler was used for amplification and incubation steps (Applied Biosystems). Libraries were quantified by Qubit^TM^ 3.0 Fluorometer (Thermo Fischer Scientific) and quality was checked using 2100 Bioanalyzer with High Sensitivity DNA kit (Agilent Technologies). Sequencing was performed in paired-end mode using NextSeq 500, HiSeq 2500, and NovaSeq 6000 sequencer (Illumina) to reach a mean of 120,000 reads per single cell.

### Data analysis of scRNA-seq

After sequencing, data were demultiplexed and mapped to the GRCh38 human reference genome and feature-barcode matrices were generated using Cell Ranger. To aggregate the naive, bystander and infected libraries of gastric unit libraries, cellranger aggr command was used with default settings and depth normalization of data. The organoid-derived monolayer infection data were analysed using RaceID, and Seurat R package in the Rstudio environment. Low-quality cells were identified based on the common quality control metrics including the number of expressed features, number of UMIs and percentage of mitochondrial genes and were removed from the downstream data analysis. The common workflow of single-cell RNA-seq data analysis was used to perform the downstream data analysis. Briefly, data were subjected to normalization, log transformation, and feature selection, and highly variable features were used to conduct dimension reduction and clustering. Highly variable features were detected using default settings and based on the PCElbowPlot (function in Seurat package), 10 first principal components were selected for dimension reduction and clustering. Clusters were defined at the resolution of 0.85, 1.4, and 0.6 for gastric units, 3D organoid and 2D monolayer cells, respectively. Afterward, identified clusters were projected on two-dimensional space using either UMAP or *t*-SNE visualizations. FindAllMarkers command from Seurat package and clustdiffgenes command from RaceID package with default settings were used to perform differential expression analysis. EnhancedVolcano was used to visualize differentially expressed genes using volcano plot.

### Cluster assignations

A defined set of established marker genes were used to define cell cluster identities (Fig. 1). We first used the ex vivo data to define the cell identities and took this as a basis to name the populations in the in vitro models. MUC6 and PGC are classical necks and chief cell markers respectively. In addition, PGA3-5 and LIPF were identified as neck cell markers^18,21,62–64^. In our data, we do not have clusters of cells that are separated by these markers. We, therefore, named the cluster expressing all these markers “Neck/chief cells”. They likely contain the 2 separated cell identities but they might not be separated at the cell numbers in our dataset. “Neck/chief cell progenitors” express the same markers at lower levels, similarly as suggested in other transcriptomic datasets^63^. “Parietal cells” were defined by classical markers ATP4B and ATP4A^21,63^. “Enteroendocrine cells” were defined by chromogranin A^21,62^.“Pit cells” were defined by high levels of MUC5AC, TFF1, GKN1 GKN2, which are markers also in histology^21,30,31^ and other transcriptomic studies^62,63^. “Pit cell progenitors” express lower levels of MUC5AC and TFF1 as well as GKN1 and GKN2^63^. “Proliferating cells” were defined by high levels of PCNA and MKI67^63,65^.

In the in vitro models, “Neck/cell progenitors” were further separated into 2 populations. Only one population expresses high levels of LIPF. We named these two populations “Neck/cell progenitor populations I and II”. The proliferating cells and early pit progenitors were not separated in the in vitro models, therefore we named this cluster “Proliferating cells and early progenitors”. In the 2D models, the “Pit cell progenitors” were further separated into 2 populations. “Pit cell progenitors I” cell cluster was marked by high MT gene expression. A similar expression signature has been observed in other transcriptomic datasets^63^. “Pit cell progenitor cells II” cluster was marked by TESC expression highlighted as pit cell marker by another transcriptomic analysis^62^.

### Mass cytometry (CyTOF)

Mass cytometry was performed as described before^66^. In brief, we used the following preconjugated antibodies (Fluidigm) as per manufacturers recommendation: p-H2AX [S139] (1:100; 3147016A, 147-Sm), cPARP (1:100; 3143011A, 143Nd). For antibodies not available as metal-conjugates, we used the Maxpar Antibody Labelling Kit (Fluidigm) according to the manufacturer’s instructions for the addition of the respective metal tags: YAP D8H1X (1:100; 14074, Cell Signaling Technologies, 150Nd), PSCA (1:50; sc-80654, Santa Cruz Technology, 159Tb).

### Urea concentration measurement

The urea concentration in the conditioned-organoid media was measured using a Urea Assay kit (ab83362, Abcam) and following the manufacturer’s instructions. Samples were collected after 24 h, centrifuged for 15 min at 1500 × *g* to remove cell debris, and diluted 1/100 in the urea kit buffer prior to quantification.

### *H. pylori* survival assay

A bacterial survival assay was performed as previously described^40^ with minor adjustments. Briefly, organoid-derived monolayers were infected with *H. pylori* strain P12 (WT, kanamycin sensitive) at MOI 1 as described above. After 6 h of infection, the culture medium was collected and centrifuged at full speed for 10 min to remove bacteria and cell debris. *H. pylori* strain P12-GFP (kanamycin-resistant) was grown to OD 0.5 as described above, harvested, washed once with PBS, and resuspended in PBS 1% BHI at a density of 1 × 10^6^ bacteria ml^−1^. An aliquot of 5 µl of the bacterial suspension was mixed with 25 µl of conditioned medium collected on the same day and incubated for 2 h at 37 °C. Then, bacterial solutions were plated onto GC agar plates as described before (supplemented with kanamycin 8 μg ml^−1^; C0378, Sigma-Aldrich) and colonies were counted.

### *H. pylori* chemotaxis assay

Chemotaxis assays were performed using the method described previously^35^ with minor changes. *H. pylori* strains were grown as described before to an OD of 0.5. Then, bacteria were harvested by centrifugation, washed once with PBS, and resuspended in PBS to a density of 1 × 10^7^ bacteria ml^−1^. An aliquot of 100 µl of the bacterial suspension was loaded into a 200 µl pipette tip. Organoid-derived monolayer conditioned media (100 µl) was drawn up through a 25 G needle into a 1 ml syringe, and the point of the syringe was submerged into the pipette tip containing the bacterial solution. After incubation horizontally for 2.5 h at 37 °C, the content of the syringe was transferred to a 1.5 ml tube, serially diluted in PBS and plated onto GC agar plates. To saturate the urea sensing potential of *H. pylori*, 500 mM urea was added to the conditioned media. In addition, syringes containing only 1 mM urea or PBS were used as a positive or negative control, respectively.

### Western blotting

Cells were washed with PBS and lysed with Laemmli’s buffer, sonicated, and separated on SDS–PAGE gels followed by electro-transfer to nitrocellulose membranes. Antibodies used for western blot analysis were: anti-CagA (1:2000, sc-25766, Santa Cruz Biotechnology), anti-P-Tyr (1:1000, sc-7020, Santa Cruz Biotechnology), anti-GKN1 (1:500, HPA047684, Atlas antibodies), anti-GKN2 (1:11,000, ab188866, Abcam), anti-MUC5AC (1:1000, MA5-12178, Thermo Fisher Scientific), anti-MUC6 (1:500, AM10120SU-N, Acris), anti-α-tubulin (1:3000, T6074, Sigma-Aldrich), anti-β-actin (1:3000, A2228, Sigma-Aldrich), anti-mouse HRP (1:10,000; NA931, GE Healthcare), anti-rabbit HRP (1:10,000; NA934, GE Healthcare). HPR signals were detected using a solution of luminol (0.25 mg ml^−1^, A8511-5G, Sigma-Aldrich) and *p*-coumaric acid (1.1 mg ml^−1^, sc-215648A, Santa Cruz Biotechnologies) with an ImageQuant LAS 4000 CCD camera (GE Healthcare). Quantification of western blots was performed with ImageJ. Uncropped images of immunoblots are included as Source Data.

### Generation of *MUC5AC* and *PSCA* knockout organoid lines

#### Plasmid constructs

Human gastric organoids were edited by the CRISPR-Cas9 system using a protocol previously described^67^. Briefly, a guide RNA (sgRNA; 5′-GCCCTCTCTCCTATCGCCCG-3′ for *MUC5AC*, 5′-TGTTGATGGCAGGCTTGGCC-3′ for *PSCA*) was designed to target the second exon of *MUC5AC* using CRISPOR^68^ and the first exon of *PSCA* using Benchling (www.benchling.com). For *MUC5AC*, overhangs were added to the gRNA sequence and cloned into AflII (R0520S, New England Biolabs) linearized gRNA_Cloning plasmid (41824, Addgene). A plasmid expressing hCas9 was acquired from Addgene (41815, Addgene). For *PSCA*, overhangs were added to the gRNA sequence and cloned into BbsI-HF (R0539S, New England Biolabs) linearized pSPCas9(BB)−2A-Puro V2.0 plasmid (62988, Addgene) which readily expresses Cas9.

#### Organoid transfection

The transfection protocol was adapted from Fujii et al.^69^. Human gastric organoids were cultured in 24-well plates as described above. Two days before the electroporation, Primocin was removed and Noggin-conditioned media was replaced with recombinant Noggin (100 ng ml^−1^, 250-38, Peprotech). A day before the electroporation, human gastric organoids were treated with 1.25% DMSO. On the day of the electroporation, organoids were shredded mechanically and made into single cells using TrypLE Express (12605036, Gibco).

A total of 5 × 10^5^ cells were resuspended in 100 μl of BTXpress buffer (45-0805, BTX Molecular Delivery Systems) supplemented with RHOKi (Y-27632; 10 μM, M1817, AbMole). Cells were electroporated with 4 μg hCas9 plasmid (41815, Addgene) and 4 μg of gRNA plasmid (41824, Addgene) or 45 µg of pSPCas9(BB)−2A-Puro V2.0 plasmid (62988, Addgene) (for *MUC5AC* KO or *PSCA* KO, respectively) in a nucleofection cuvette (EC-002S, Nepagene) using the NEPA 21 Super Electroporator (Nepagene) with following settings: Poring pulse (175 V, pulse length—5 ms, pulse interval—50 ms, number of pulses—2, decay rate—10% and positive polarity), Transfer pulse (20 V, pulse length—50 ms, pulse interval—50 ms, number of pulses 5, decay rate—40% and positive/negative polarity). After electroporation, cells were washed with Opti-MEM (31985070, Gibco) containing RHOKi (Y-27632; 10 μM, M1817, AbMole) and seeded in Matrigel (356231, Corning). The organoid medium was supplemented with RHOKi (Y-27632; 10 μM, M1817, AbMole), nicotinamide (10 mM, 72340, Sigma Aldrich), prostaglandin E2 (10 mM, 2296, TOCRIS bioscience) and GSK-3 inhibitor (CHIR99021, 1386, Axon MedChem). CHIR99021 was removed after 24 h. *MUC5AC*-KO organoids were selected with puromycin (2 µg ml^−1^, sc-108071B, Santa Cruz Biotechnologies) for 48 h five days after electroporation. PSCA-KO organoids were selected with 1 μg ml^−1^ puromycin on days 4–7 after electroporation. Individual organoids were split and seeded in 25 µl Matrigel (356231, Corning). Organoid lines were maintained and expanded until molecular characterization. Genomic DNA from potential KO organoid lines was isolated and used to amplify a fraction of *MUC5AC* or *PSCA* containing the gRNA (5′-GTGGTCTGGTCCCACTATGCTG-3′, 5′-GTGGAGGGTGGAATCTGACA-3′ for *MUC5AC*, 5′-ATGGCCCTGGGTAGGCTCTGTC-3′, 5’-GAAGCTGCAGTGCTGGGACTGG-3′ for *PSCA*). Sanger sequencing was used to confirm proper KO at the targeted locus. *MUC5AC* KO phenotype was confirmed by western blot and immunostaining. *PSCA* KO phenotype was confirmed by flow cytometry.

### Statistical analysis

Data are presented as mean ± standard deviation (SD), with the number of experiments performed and organoid lines indicated in figure legends. Statistical analysis was performed using Prism Software (v6.01, GraphPad). Statistical significance was defined as *p*  <  0.05. Statistical analyses are detailed in Supplementary Data 6.

### Reporting summary

Further information on research design is available in the Nature Research Reporting Summary linked to this article.

## Supplementary information

Supplementary Information

Supplementary Dataset 1

Supplementary Dataset 2

Supplementary Dataset 3

Supplementary Dataset 4

Supplementary Dataset 5

Supplementary Dataset 6

Supplementary Movie 1

Reporting Summary

## Data Availability

scRNA-seq data from ex vivo gastric units, gastric organoids and 2D organoid-derived monolayers (naïve, bystander and *H. pylori*-infected) samples are publicly available in the GEO repository database under the accession number GSE167561. All other relevant data are available from the corresponding authors upon request. Source data are provided in this paper.

## Source data

Source Data
