# Supplementary material for: Helicobacter pylori shows tropism to gastric differentiated pit cells dependent on urea chemotaxis
Source: Nat Commun. 2022 Oct 5;13:5878. doi: 10.1038/s41467-022-33165-4 (PMC9535007; doi:10.1038/s41467-022-33165-4)
Supplement: Supplementary file 1 — Supplementary Information [file 41467_2022_33165_MOESM1_ESM.pdf]

Helicobacter pylori shows tropism to gastric differentiated pit cells dependent on urea chemotaxis.

Carmen Aguilar, Mindaugas Pauzuolis, Malvika Pompaiah, Ehsan Vafadarnejad, Panagiota Arampatzi, Mara Fischer, Dominik Narres, Mastura Neyazi, Özge Kayisoglu, Thomas Sell, Nils Blüthgen, Markus Morkel, Armin Wiegering, Christoph-Thomas Germer, Stefan Kircher, Andreas Rosenwald, Antoine-Emmanuel Saliba, Sina Bartfeld.

Supplementary Information

SUPPLEMENTARY FIGURES

Supplementary Figure 1

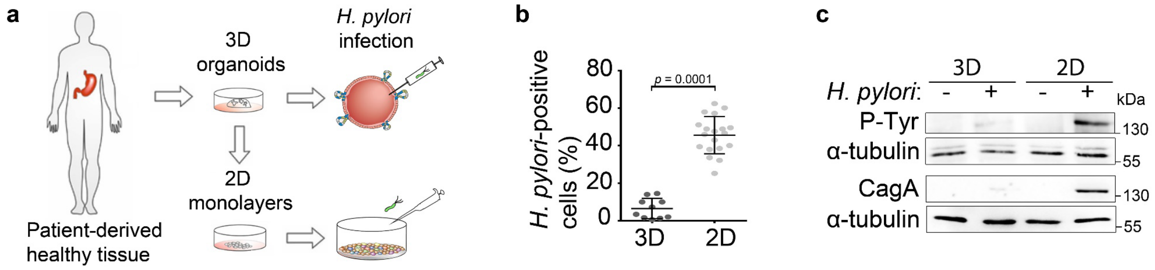

Supplementary Figure 1. ***H. pylori* attaches and translocates CagA into 2D monolayers more efficiently than to 3D organoids. a,** Scheme of the experimental setup. 3D organoids were generated from normal gastric tissue from human donors and infected by microinjection with *H. pylori* or used to generated 2D monolayers that were then also infected. **b,** Flow cytometry analysis of *H. pylori* adhesion to 2D monolayers or 3D organoid model. **c,** Western blot for CagA total protein (CagA) and translocated CagA (Phosphorylated CagA, P-Tyr) in *H. pylori*-infected 2D monolayers and 3D organoids. Panel b showing data of organoid lines derived from 3 donors (b) and 3 (3D condition) or 6 (2D condition) independent experiments. Data in panel c showing representative results from data of 3 organoid lines and 1 independent experiment. Results are shown as mean ± SD (b). Statistical analysis of data in panel b was performed using a two-tailed Student's t-test. Source data are provided as a Source Data file.

Supplementary Figure 2

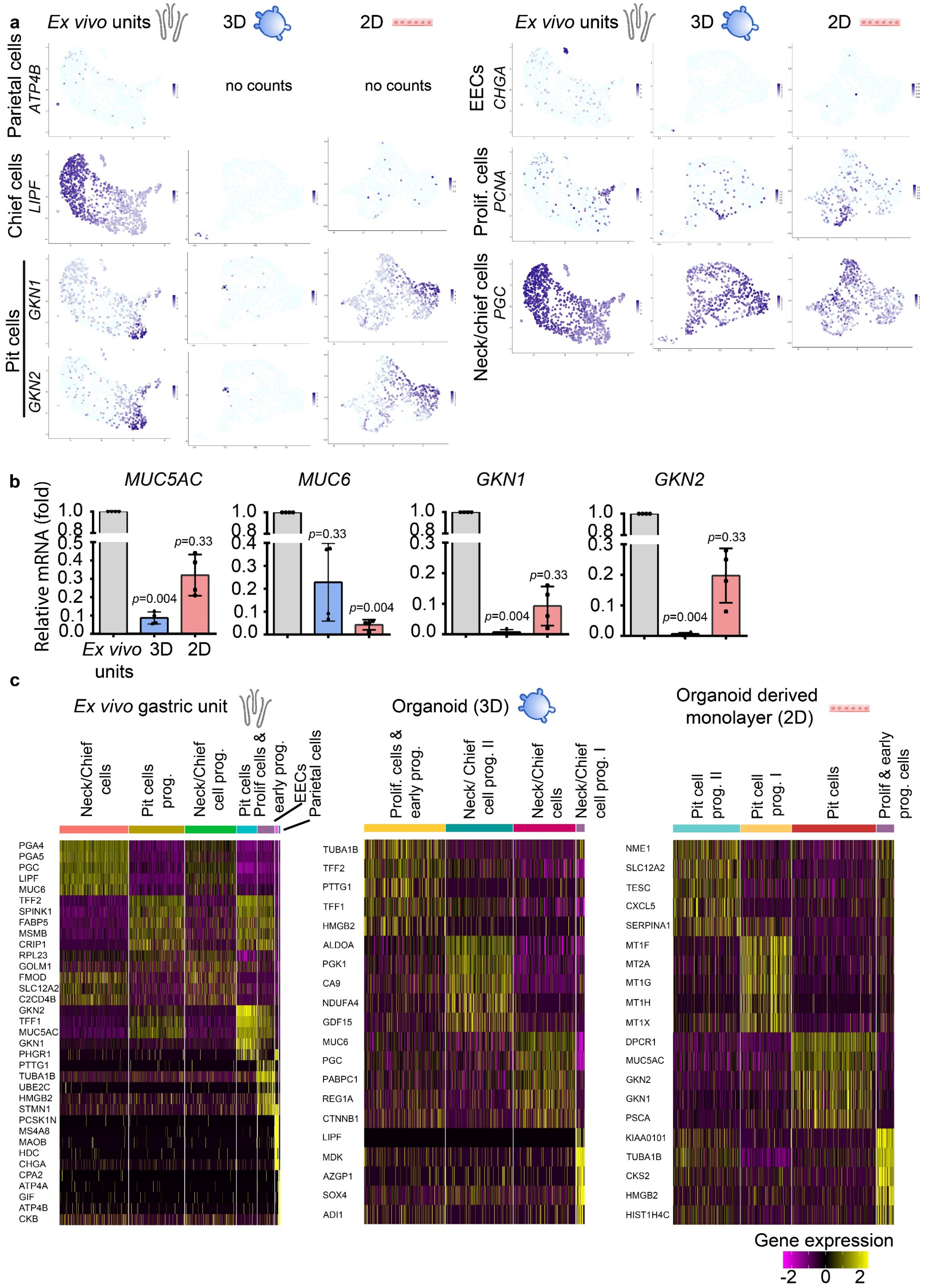

Supplementary Figure 2. **2D monolayers and 3D organoids are composed of different cell types. a,** Expression of known markers specific for the different gastric epithelial cell types colour-coded and projected on top of the UMAPs as in Fig. 1a. **b,** qRT-PCR analysis of *MUC5AC*, *MUC6*, *GKN1* and *GKN2* mRNA in *ex vivo* gastric units, organoids and 2D monolayers. Results are normalized against the *ex vivo* gastric units. **c,** Heatmaps showing differential gene expression of the top 5 genes per cluster in the *ex vivo* gastric unit, 3D organoid and organoid-derived monolayer datasets. Marker genes were identified using Seurat FindAllMarkers function (Wilcoxon Rank-Sum test, min.pct = 0.25, logfc.threshold = 0.25). Results in b are shown as mean ± SD of organoids lines generated from 4 donors and 1 independent experiment. Statistical analysis of data in panel b was performed using the Kruskal-Wallis test with Dunn's multiple comparisons test.

Supplementary Figure 3

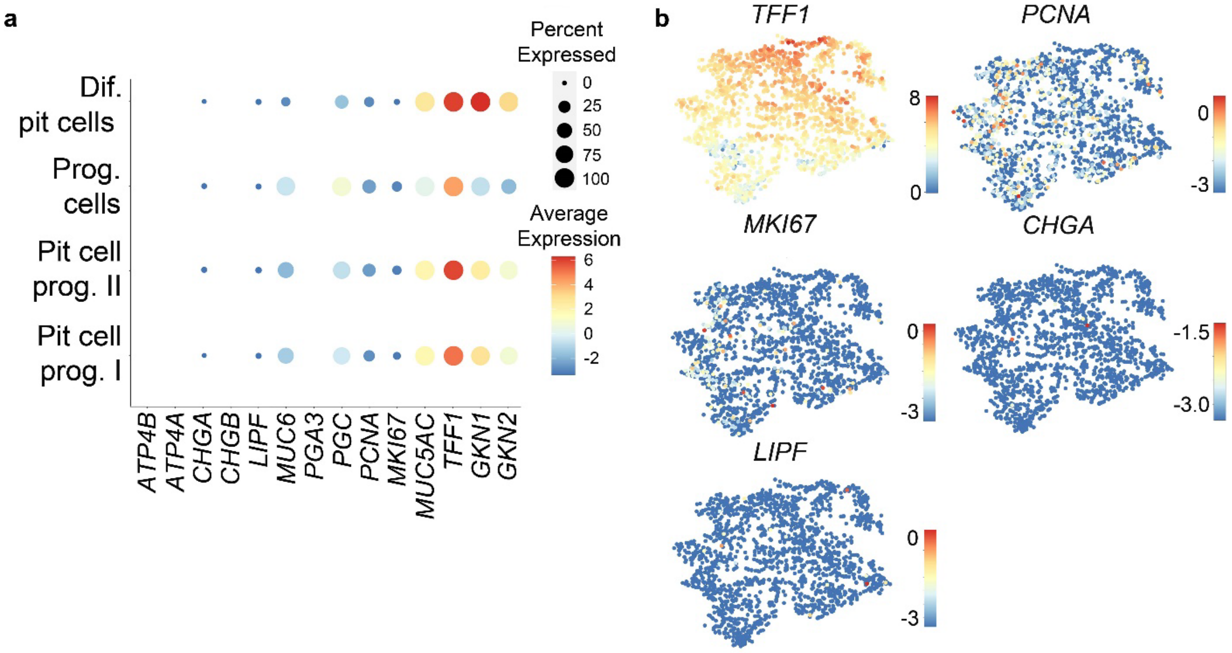

Supplementary Figure 3. **scRNA-seq profiling of *H. pylori* infected 2D monolayers. a,** Dot plot showing the expression of cell type specific marker genes within the clusters. **b,** Expression of known markers specific for the different gastric epithelial cell types, colour-coded and projected on top of the t-SNE as in Fig. 2c.

Supplementary Figure 4

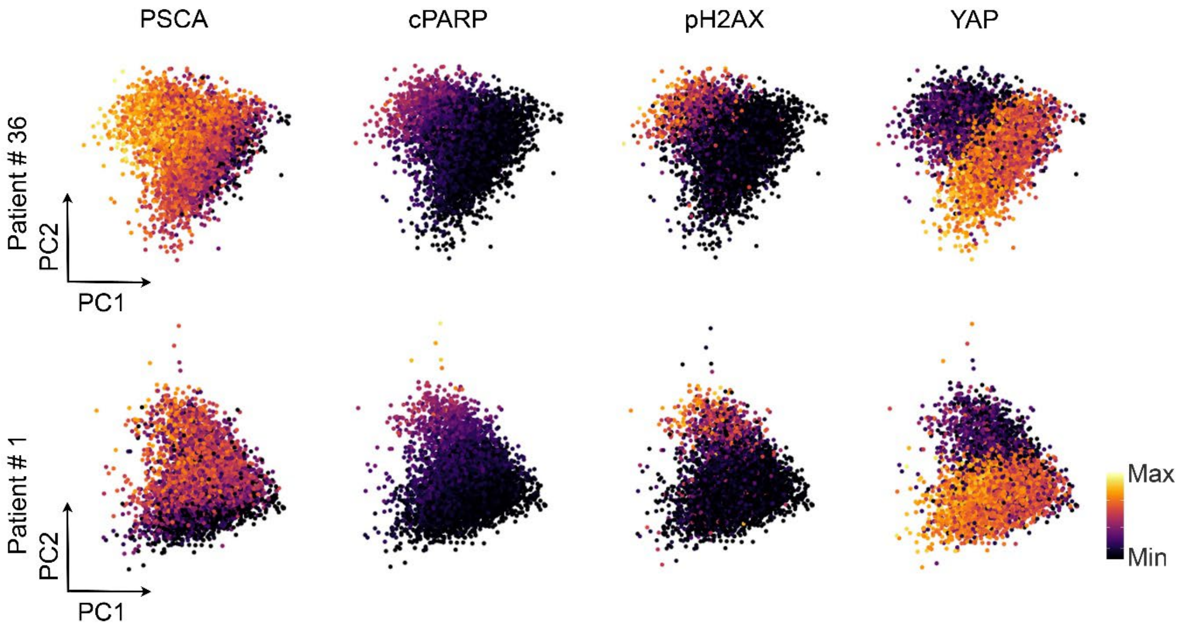

Supplementary Figure 4. **PSCA expression localizes with apoptotic markers.** Protein levels of PSCA, cleaved PARP, phosphorylated H2AX and YAP in 2D naïve monolayers (Patient #36 and #1) measured by mass spectrometry. Every dot represents an individual cell and colours denote intensity of the signal.

Supplementary Figure 5

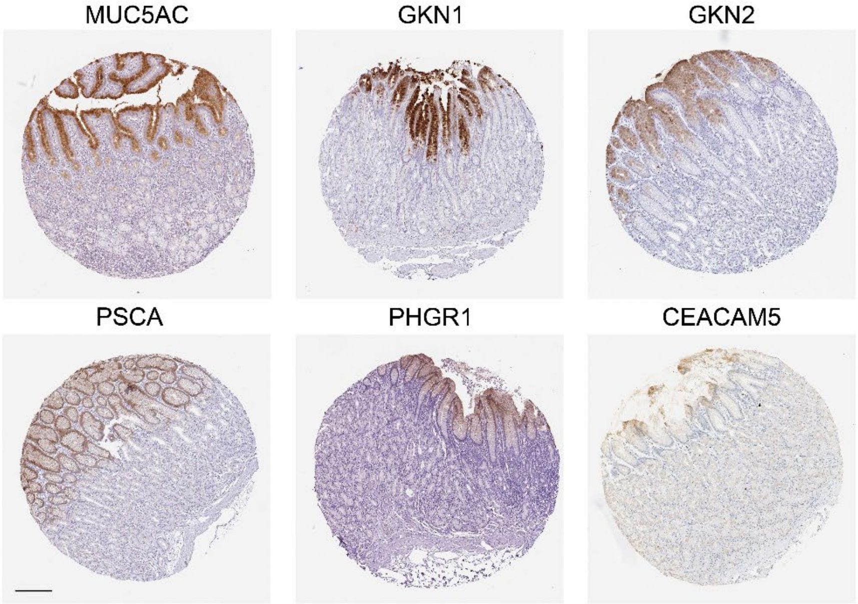

Supplementary Figure 5. ***H. pylori* target cell marker proteins localize to the pit region of gastric units.** Immunohistochemistry analysis of MUC5AC, GKN1, GKN2, PSCA, PHGR1 and CEACAM5 protein expression in human gastric tissue (Data from The Human Protein Atlas Database, See Methods for more detail). Scale bar: 200 µm.

Supplementary Figure 6

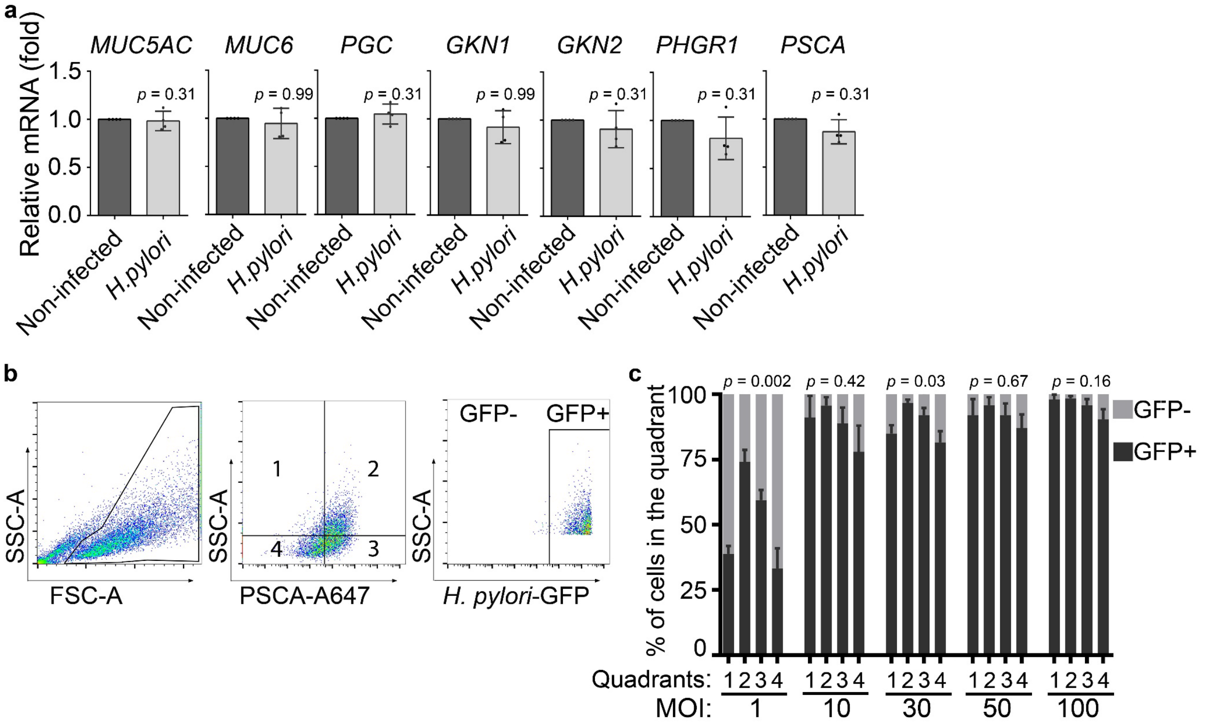

Supplementary Figure 6. ***H. pylori* infection of 2D monolayers do not regulate gene expression of the target cell population markers and *H. pylori* tropism is not observed at high MOIs. a,** qRT-PCR analysis of *MUC5AC*, *MUC6*, *PGC*, *GKN1*, *GKN2*, *PHGR1* and *PSCA* mRNA upon infection with *H. pylori*. Results are normalized against control mock-infected. **b,** Representative flow cytometry analysis of *H. pylori*-infected cells and PSCA protein in 2D monolayers. Cells were divided into four quadrants based on size and PSCA signal, and subsequently *H. pylori*-infected cells were quantified in each quadrant. A647: Alexa-Fluor647. **c,** Flow cytometry analysis of *H. pylori*-infected cells per quadrant (as shown in panel b). Results are shown as mean ± SD of organoid lines generated from 4 donors (a) and 3 donors (c), and 1 (a) or 3 independent experiments (c). Data in panel b is representative of results from organoid lines from 3 donors. Statistical analysis of data in panel a was performed using two-tailed Student's t-test, and panel c with Kruskal-Wallis test with Dunn's multiple comparisons test.

Supplementary Figure 7

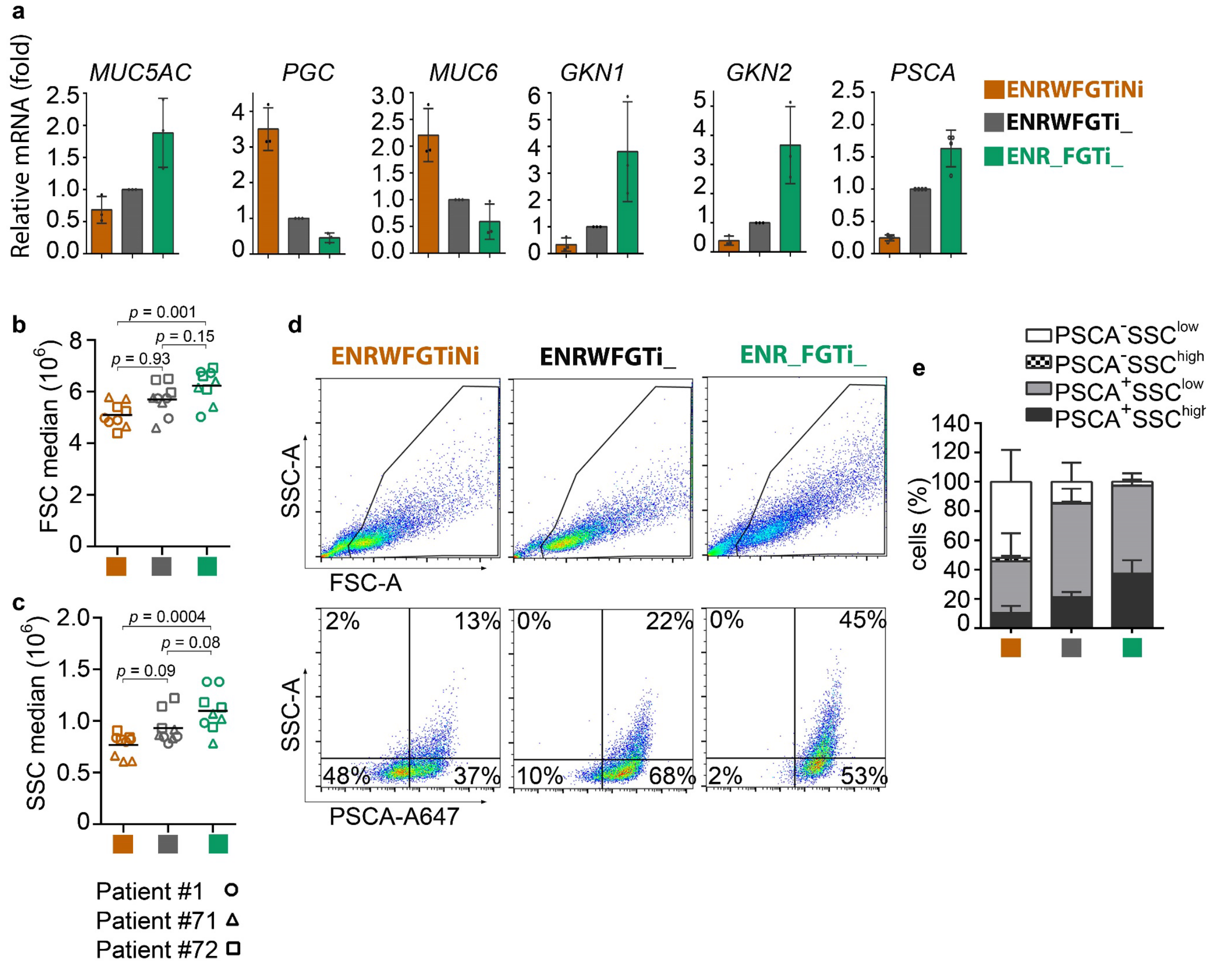

Supplementary Figure 7. **Directed differentiation of 2D monolayers allows enrichment or depletion of differentiated pit cell population. a,** qRT-PCR quantification of markers specific for pit cells (*MUC5AC*), neck cells (*MUC6*), chief cells (*PGC*) and the identified markers for differentiated pit cells (*GKN1*, *GKN2* and *PSCA*) in 2D monolayers upon differentiation according to the scheme in Fig. 4a. **b-c,** Median FSC (**b**) and SSC (**c**) values of 2D monolayer cells grown in the different conditions. **d,** Representative flow cytometry analysis of PSCA protein in 2D monolayers grown in the different conditions. A647: Alexa-Fluor647. **e,** Quantification of PSCA protein measured by flow cytometry. Results are shown as mean ± SD (a and e) from organoid lines generated from 3 (a) and 4 (e) donors, and 1 (a) or 3 (e) independent experiments. In panel b-c organoid lines from 3 individual donors are shown as symbols (patient #1-circle, #71-triangle, and #72-square), mean values with horizontal lines (3 independent experiments). Data in panel d is representative of results from organoids of 4 donors. Statistical analysis of data in panel a was performed using Kruskal-Wallis test with Dunn's multiple comparisons test, and in panel b-c with one-way ANOVA with Tukey's multiple comparisons test.

Supplementary Figure 8

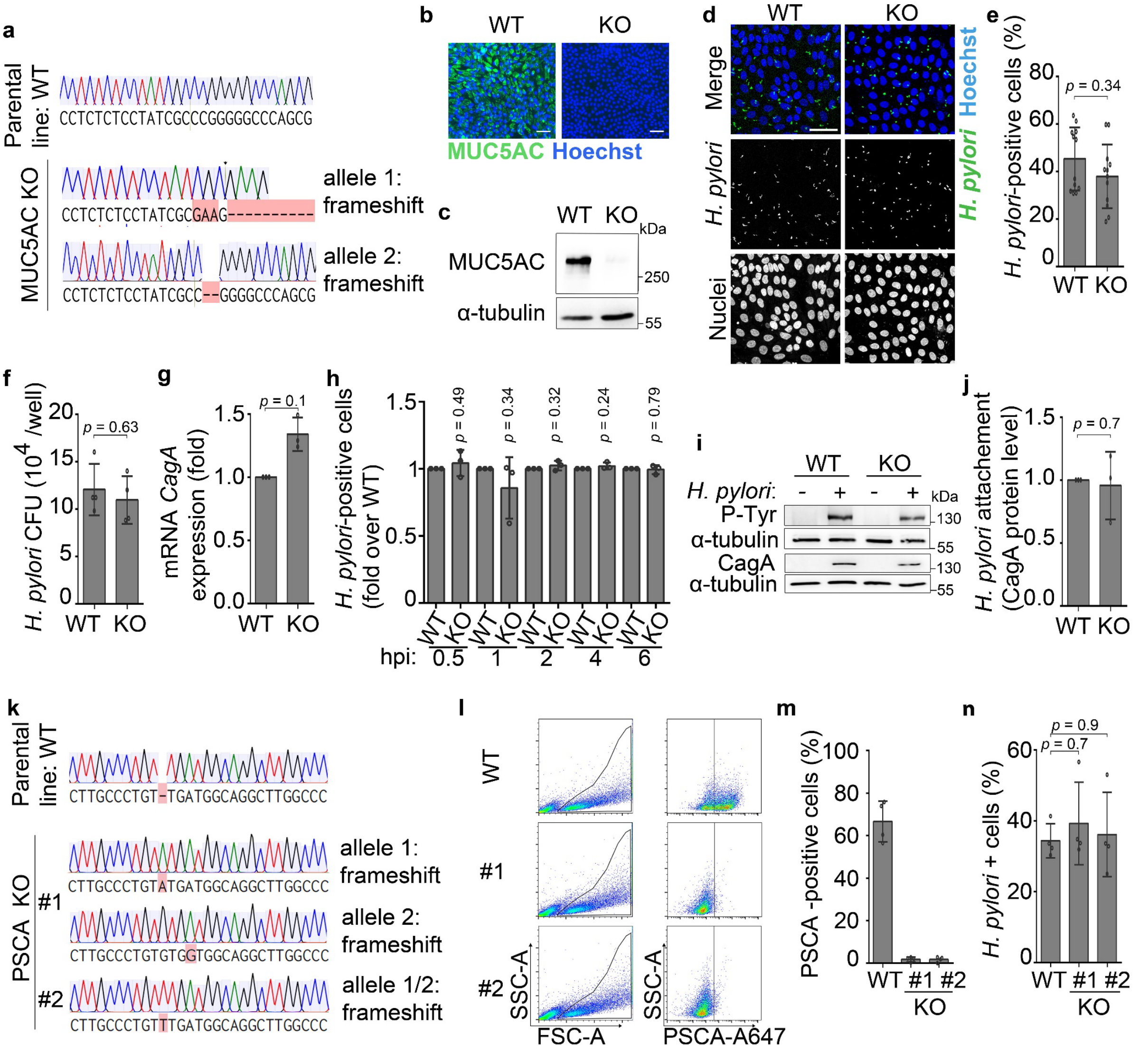

Supplementary Figure 8. ***H. pylori* adhesion is independent of *MUC5AC* and *PSCA* expression in organoid-derived monolayers. a-c,** Validation of a MUC5AC KO gastric organoid line generated via CRISPR/Cas9 editing: **a,** Sanger sequencing indicates bi-allelic MUC5AC KO. **b-c,** Immunofluorescence (**b**) and western blot (**c**) analysis of MUC5AC parental WT and KO organoid lines. Scale bar: 25µm. **d,** Representative images of parental WT and *MUC5AC* KO organoid lines infected with GFP-expressing *H. pylori* (green) at MOI 1 for 6 h. Scale bar: 50 µm. **e-g,** Quantification of adhered bacteria to the parental WT and MUC5AC KO organoid lines measured by flow cytometry (**e**) CFU assay (**f**) and qRT-PCR of *CagA* (**g**). **h**, Quantification *H. pylori* adhesion to the parental WT and MUC5AC KO organoid-derived monolayers during a time course of 6 hours. **i,** Representative western blot for CagA total protein (CagA) and translocated CagA (phosphorylated CagA, P-Tyr) in the parental WT and MUC5AC KO lines upon infection. **j,** Quantification of bacterial attachment (CagA over host alpha-tubulin) by western blot. Results in g, h, and j were normalized against parental WT. **k-m**, validation of two PSCA KO gastric organoid lines generated via CRISPR/Cas9 editing: **k**, Sanger sequencing shows a frameshift of the coding sequence in both KO lines. **l-m**, representative representation (**l**) and analysis of PSCA levels quantified by flow cytometry (m). **n**,

Quantification of adhered bacteria to the parental WT or PSCA KO organoid-derived monolayers. Results are shown as mean ± SD of at least 3 independent experiments (panel b-j and l-n). Data in panel d, i and l are representative of results from 3 independent experiments. Statistical analysis of data in panel e-g and j was performed using Two-tailed Student's test, panel h with a multiple t-test corrected for multiple comparisons using the Holm-Sidak method and, in panel m-n with Kruskal-Wallis test with Dunn's multiple comparisons test. Source data are provided as a Source Data file.

Supplementary Figure 9

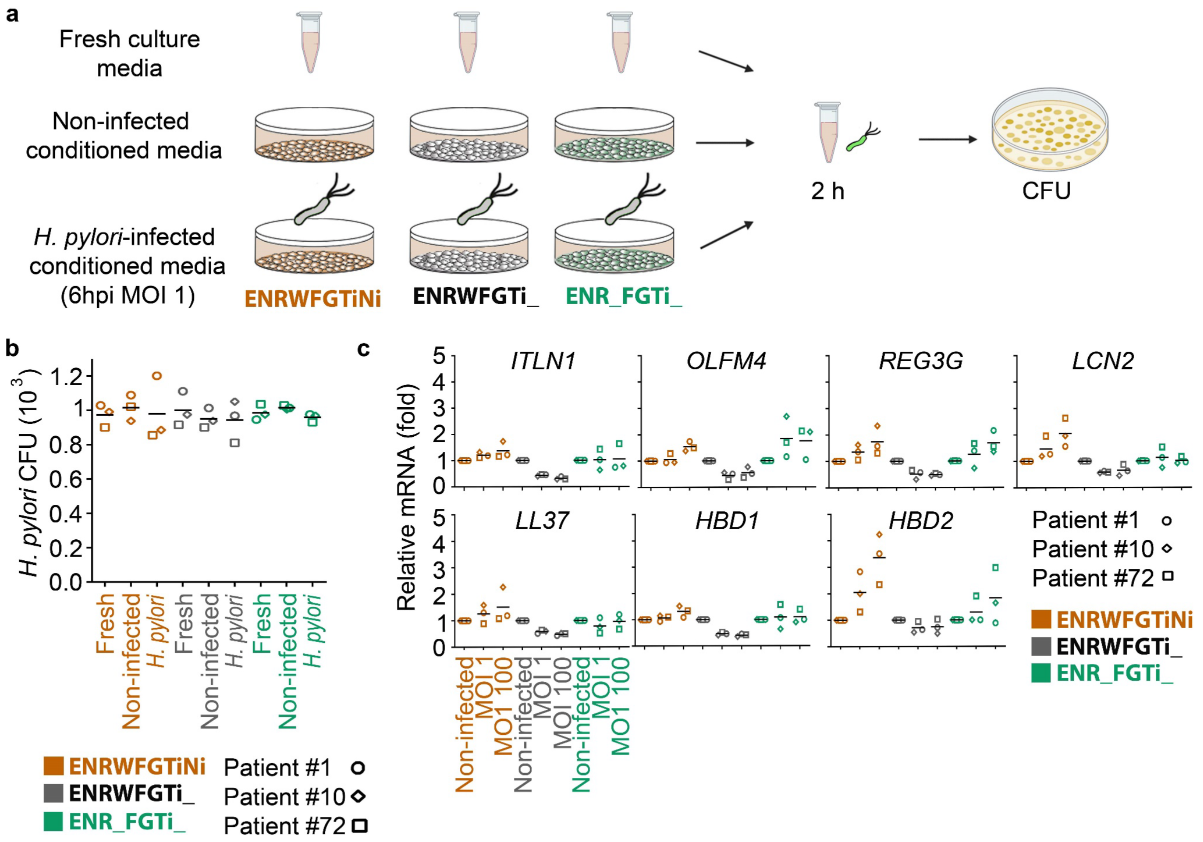

Supplementary Figure 9. Antimicrobial response of gastric organoid derived-monolayers has no effect on *H. pylori* survival. a, Scheme of the experimental setup. *H. pylori* was incubated with conditioned media from mock- and *H. pylori*-infected cells after 6 hpi for 2 h and then serially plated. Fresh culture media was used as control. **b,** Quantification of bacterial survival after incubation with fresh, non-infected or *H. pylori*-infected cells conditioned media. **c,** qRT-PCR quantification of antimicrobial genes upon infection with *H. pylori*. Data from organoid lines from individual donors are shown as symbols (patient #1-circle, #10-diamond, and #72-square), mean values with horizontal lines. Data in panel b and c is representative of results from 3 independent experiments with organoid lines derived from 3 donors. Statistical analysis of data in panel b was performed using one-way ANOVA with Tukey's multiple comparisons test, and in panel c with Kruskal-Wallis test with Dunn's multiple comparisons test.
